# The Impact of Travel Distance to Delayed Presentation and Follow-up Attendance of Retinal Detachment Cases in Surabaya, Indonesia

**DOI:** 10.14789/jmj.JMJ21-0024-OA

**Published:** 2022-02-04

**Authors:** SAULI ARI WIDJAJA, YOSHIMUNE HIRATSUKA, KOICHI ONO, MUHAMMAD FIRMANSJAH, WIMBO SASONO, AKIRA MURAKAMI

**Affiliations:** 1 Department of Ophthalmology, Juntendo University Graduate School of Medicine, Tokyo, Japan; 1 Department of Ophthalmology, Juntendo University Graduate School of Medicine, Tokyo, Japan; 2 Department of Ophthalmology, Juntendo Tokyo Koto Geriatric Medical Center, Tokyo, Japan; 2 Department of Ophthalmology, Juntendo Tokyo Koto Geriatric Medical Center, Tokyo, Japan; 3 Department of Ophthalmology, Faculty of Medicine Universitas Airlangga/Dr. Soetomo General Academic Hospital, Surabaya, East Java, Indonesia; 3 Department of Ophthalmology, Faculty of Medicine Universitas Airlangga/Dr. Soetomo General Academic Hospital, Surabaya, East Java, Indonesia

**Keywords:** retinal detachment, delayed presentation, symptom onset, follow-up attendance, health system, accessible

## Abstract

**Objectives:**

To assess the delayed presentation of Retinal Detachment (RD), its association from travel distance to the referral hospital (TDH), the period from symptom onset to consultation (SO-C), Proliferative vitreoretinopathy (PVR) severity, and 6 months follow-up attendance (6mo-FA).

**Method:**

A retrospective review based on medical records. Age, sex, initial best-corrected visual acuity (BCVA), TDH, SO-C, PVR type, and 6mo-FA were recorded. Multivariable ordered logistic regression was used to analyze the association between TDH and SO-C, and SO-C and PVR severity. Multivariable logistic regression was used to analyze 6mo-FA according to TDH. Multiple linear regression was used to assess the association between initial BCVA and TDH. Age and sex were included in all multivariable adjustments.

**Results:**

A total of 387 patients had RD with 59.2% predominantly males and the mean age±SD was 46.3±13.9 years. The initial BCVA of less than 3/60 was 81.1%. The averages of SO-C and TDH were 183.5±456 days and 160.9±364 km, respectively. The TDH of more than 120 km distance was significantly associated with longer SO-C (adjusted OR 1.78; CI 95% 1.09-2.92). PVR was noted in 17.6% of patients. The SO-C of 31-60 days was significantly associated with PVR severity (adjusted OR 4.28; CI 95% 1.47-12.51). The TDH of more than 120 km distance was significantly associated with 6mo-FA (adjusted OR 0.46; CI 95% 0.27-0.93).

**Conclusions:**

Long TDH was significantly associated with a longer period from symptom onset to consultation and 6mo-FA. Hence, accessible eye care is essential to refer RD cases in a timely fashion.

## Introduction

Permanent blindness is often caused by retinal disease, glaucoma, and other disorders that present late or with insufficient care^1)^. Furthermore, delay in bringing retinal detachment (RD) cases to retinal specialists tends to be a trend in developing countries, the treatment of which has been a minor priority^2)^. Lack of education, access, and inadequate healthcare coverage are probable contributing factors^3)^. At many stages in the healthcare phase, delays between first symptoms and surgical repair may occur, which can be either from the doctor or the patient^4)^. In one study from Indonesia, childhood RD was revealed to often arrive late for assessment^5)^. Other studies pointed out that patients are more likely to delay the examination and appear as complex RD^2, 3)^. This is similar to other findings that suggest that later and severe RD cases appear to emerge from more deprived regions. This pattern has significant effects on the final visual prognosis^6)^.

Retinal detachment can be estimated at 17,500 to 25,000 new cases annually in Indonesia. However, the national annual report for the number of RD surgeries is not provided^1)^. In Indonesia, approximately 1,600-2,800 vitreoretinal doctors are expected to serve 230 million people. Given the small number of VR surgeons in Indonesia, especially now that the population has reached 270 million, treating VR diseases requires not only timely intervention but also easier access to referral healthcare centers and affordability^1)^. Healthcare access is also impacted by transportation, especially in rural regions where travel distances are long and alternative forms of transportation are few^7, 8)^.

While retinal detachment has added to the burden of blindness in Indonesia, few studies have directly discussed their epidemiology and effects on the scale of retinal diseases. In this paper, we will study what has been learned about RD in developing countries with limited facilities and uneven vitreoretinal surgeon distribution. We would like to identify the situation particularly at Dr. Soetomo General Academic Hospital (DSGAH) as one of the national referral hospital in Indonesia that located in East Java. East Java has two other hospitals that are equivalent to DSGAH, however comprehensive vitreoretinal facilities are inadequate. The purpose of this research was to study discrepancies between travel distance to the referral hospital (TDH) as one of the socioeconomic factors that impact the referral pathway. We hypothesized that patients living far from the referral hospital will be more likely to seek treatment late, which would lead to more severity and having unfavorable compliance in completing follow-up treatment. As such, late referral, the longer period from symptom onset to consultation (SO-C), and severe retinal proliferation occurrence might impact the final visual acuity (VA). These findings will hopefully provide the data to enhance access to eye care, lead to better screening programs in peripheral areas, and raise awareness not only for health workers but also the people at risk so that early identification and diagnosis can be achieved as timing is a critical factor in RD management.

## Methods

## Material and Methods

A retrospective medical chart review was conducted at the Ophthalmology Out Patient Department (OPD) of Dr. Soetomo General Academic Hospital (DSGAH), Surabaya, East Java, Indonesia from 2013-2017. DSGAH is one of two other national referral hospital in East Java that serves as a teaching hospital and a referral center for the east part of Indonesia. The review was performed on all cases diagnosed with RD by the consultant in the vitreoretinal unit. Approval to review the medical record was obtained from DSGAH Institutional Review Board (IRB) under the number 0977/ KEPK/ II/ 2019. All the procedures performed in this study were in accordance with the ethical standards of the Declaration of Helsinki and the IRB. The IRB committee has been informed of the objective of the study and how the data will be used. As this study presented no risk to the participants and did not violate individual rights, thus access to medical records has been granted without informed consent needed.

Demographic details including age, sex, travel distance to the referral hospital (TDH), the period from symptom onset to consultation (SO-C), Proliferative vitreoretinopathy (PVR) severity, and 6 months follow-up attendance (6mo-FA) were obtained. The TDH was determined by the distance between the place of origin (where the patient lives) and DSGAH Surabaya in kilometer (km) using an online distance calculator. The SO-C was determined by patients' subjective reports in the medical record, which defines the period between the first onset of retinal detachment symptoms such as floaters, photopsia, visual field defect or vision disturbances, and the first examination at OPD.

The presenting BCVA and 6 months post-operative BCVA from the affected eye were recorded from Snellen's charts examination and converted to LogMAR for analysis. Proliferative vitreoretinopathy (PVR) type according to the Updated Retina Society Classification (1991)^9)^ and macula condition (macula-on or macula-off) were retrieved. Additionally, 6mo-FA was obtained from the availability of the data in the medical records, including anatomical retinal reattachment in 6 months post-operative and 6 months postoperative BCVA.

## Statistical Analysis

Travel distance to the referral hospital (TDH) was classified into 5 groups, less than or equal to 30 km, 31-60 km, 61-90 km, 91-120 km, and more than 120 km. SO-C was categorized into 5 groups, less than or equal to 7 days, 8-14 days, 15-30 days, 31-60 days, and more than 60 days. Further, PVR type was classified as type 0 (No PVR), type 1 (PVR grade A and grade B), and type 2 (PVR grade C). The results were reported as mean and standard deviation for quantitative variables and percentage for categorical variables. Univariate and multivariable ordered logistic regression models were used to estimate the crude and adjusted odds ratios (ORs) and their 95% confidence interval (CIs) for SO-C according to TDH, and PVR severity according to SO-C. The Brant test of parallel regression assumption was used to test whether the relationship between each pair of outcome groups was the same. A nonsignificant test statistic provided evidence that the parallel regression assumption had not been violated. We used univariate and multiple linear regression analysis to estimate the crude and adjusted coefficients and their 95% CIs to assess the association between initial BCVA and TDH. Furthermore, the univariate and multivariable logistic regression model was used to estimate the crude and adjusted ORs and their 95% CIs for 6mo-FA according to TDH. In all multivariable adjustments, age and sex were included. IBM SPSS version 23 and Stata version 15 (StataCorp, College Station, TX, USA) were used to perform all the statistical analyses. P values <0.05 were considered statistically significant.

## Results

Retinal Detachment (RD) found in 387 patients consisted of 229 (59.2%) males and 158 (40.8%) females, with the age range of 7-76 years (mean± SD: 46.3±13.9 years) (Table 1). The majority of the patients (359; 92.8%) came from Java Island. Of the 387 patients, 172 (44.4%) came from a distance of less than or equal to 30 km from DSGAH, and 94 (24.3%) were from a distance of more than 120 km (Table 1). The mean ± SD travel distance to the referral hospital (TDH) was 160.9±364 km with a median of 52 (10-2819) km. The mean ± SD period from symptom onset to consultation (SO-C) was 183.5±456 days with a median of 30 (2-3775) days. When broken down into categories, most patients came with a SO-C of more than 60 days (128; 33.1%) (Table 1). After the adjustment for age and sex, compared to the TDH of less than or equal to 30 km, only the TDH of more than 120 km distance was significantly associated with a longer SO-C (adjusted OR 1.78; 95% CI 1.09-2.92) (Table 2). The result of the brant test for proportional odds assumption was not statistically significant (p = 0.31), indicating that this method of analysis was appropriate for this study.

**Table 1:** Demographic of retinal detachment patients in Surabaya, Indonesia. (N=387)

Demography Characteristics	Value
Sex (n,%)	
Male	229 (59.2)
Female	158 (40.8)
Age (years)	
Mean ± SD	46.3±13.9
Median (range)	49 (7-76)
Place of Origin (n,%)	
Java Island	359 (92.8)
Outside of Java Island	25 (6.5)
Unknown	3 (0.8)
Travel distance to the referral hospital (TDH) (kilometers)	
Mean ± SD	160.9±364
Median (range)	52 (10-2819)
TDH Categories (n,%)	
≤30 km	172 (44.4)
31-60 km	45 (11.6)
61-90 km	32 (8.3)
91-120 km	41 (10.6)
>120 km	94 (24.3)
unknown	3 (0.8)
Period from symptom onset to consultation (SO-C) (days)	
Mean ± SD	183.5±456
Median (range)	30 (2-3775)
SO-C Categories (n,%)	
≤7 days	61 (15.8)
8-14 days	46 (11.9)
15-30 days	74 (19.1)
31-60 days	32 (8.3)
>60 days	128 (33.1)
Unknown	46 (11.9)
PVR grade (n,%)	
Type 0 (No PVR)	225 (58.1)
Type1 (Grade A-B)	39 (10.1)
Type 2 (Grade C)	29 (7.5)
unknown	94(24.3)
Macular condition (n,%)	
On	63 (16.3)
Off	274 (70.8)
Unknown	50 (12.9)

**Table 2:** Odds ratios of travel distance to the referral hospital (TDH) on the period from symptom onset to consultation (SO-C) by multivariable ordered logistic regression.

TDH	Crude Odds Ratio	95% CI	P-value	Adjusted Odds Ratio	95% CI	P-value
≤30km	reference			reference		
31-60 km	1.32	0.71-2.49	0.38	1.35	0.71-2.56	0.35
61-90 km	0.66	0.33-1.33	0.25	0.67	0.33-1.35	0.26
91-120 km	1.49	0.78-2.86	0.23	1.49	0.77-2.89	0.23
More than 120 km	1.73	1.06-2.81	0.03	1.78	1.09-2.92	0.02

Proliferative vitreoretinopathy (PVR) prior to surgery was noted in 17.6% (13.9-21.7) patients consisting of PVR grade A-B (39; 10.1%) and C (29; 7.5%), while most of the cases (225; 58.1%) were without PVR. After the adjustment for age and sex, compared to the SO-C of less than or equal to 7 days, only the SO-C of 31-60 days was significantly associated with advanced PVR type (adjusted OR 4.28; 95% CI 1.47-12.51) (Table 3). The result of the brant test for proportional odds assumption was not statistically significant (p = 0.34), indicating that this method of analysis was appropriate for this study. Macula off presented in 274 (70.8%: 95% CI 66.0-75.3) patients.

**Table 3:** Odds ratios of the period from symptom onset to consultation (SO-C) on PVR severity by multivariable ordered logistic regression.

SO-C	Crude Odds Ratio	95% CI	P-value	Adjusted Odds Ratio	95% CI	P-value
≤7 days	reference			reference		
8-15 days	1.86	0.71-4.87	0.21	1.75	0.66-4.65	0.26
16-30 days	1.20	0.46-3.15	0.70	1.22	0.45-3.27	0.70
31-60 days	4.02	1.39-11.58	0.01	4.28	1.47-12.51	0.01
> 60 days	0.99	0.42-2.36	0.99	1.02	0.43-2.44	0.96

Table 4 shows the visual acuity at the time of the initial examination and after the 6-month follow-up. The initial BCVA of less than 3/60 (LogMAR 1.3) was most encountered (81.1%). The mean initial BCVA was LogMAR 1.96±0.58 (range: LogMAR 0.0-3.0). The mean BCVA 6 months after surgery was LogMAR 1.42±0.59 (range: LogMAR 0.1-2.7). Table 5 presents the results of linear regressions to evaluate the association between initial BCVA and TDH. After the adjustment for age and sex, no significant association was evident between initial BCVA and TDH.

**Table 4:** Initial BCVA and 6–month follow-up BCVA.

VA Category (n,%)	Initial BCVA	6 mo follow-up BCVA
> 6/18 (< LogMAR 0.5)	11 (2.8%)	5 (1.3%)
6/18-6/60 (LogMAR 0.5-1)	23 (5.9%)	13 (3.4%)
6/60-3/60 (LogMAR 1-1.3)	20 (5.2%)	19 (4.9%)
< 3/60 (> LogMAR 1.3)	314 (81.1%)	44 (11.4%)
Unknown	19 (4.9%)	306 (79.1%)
VA mean and median (LogMAR)		
Mean ± SD	1.96 ± 0.58	1.42 ± 0.59
Median (range)	2.3 (0.0-0.3)	1.48(0.1-2.7)

**Table 5:** Coefficients of travel distance to the referral hospital (TDH) on initial BCVA by multiple linear regression.

TDH	Crude coefficients	95% CI	P-value	Adjusted coefficients	95% CI	P-value
≤30km	reference			reference		
31-60 km	0.01	-0.19-0.20	0.94	0.02	-0.17-0.22	0.81
61-90 km	-0.06	-0.29-0.17	0.61	-0.05	-0.27-0.18	0.69
91-120 km	0.13	-0.07-0.33	0.19	0.15	-0.05-0.36	0.14
≥120 km	-0.11	-0.25-0.04	0.16	-0.11	-0.26-0.04	0.16

Of the 387 patients, 72 (18.6 %: 95% CI 14.9-22.8) could be followed up for 6 months. Table 6 shows the results of logistic regressions to evaluate the association between TDH and 6 months follow-up attendance (6mo-FA). Again, only the TDH of more than 120 km was significantly associated with 6mo-FA (adjusted OR 0.46; CI 95% 0.27-0.93).

**Table 6:** Odds ratios of travel distance to the referral hospital (TDH) on 6 months follow-up attendance (6mo-FA) by multivariable logistic regression.

TDH	Crude Odds Ratio	95% CI	P-value	Adjusted Odds Ratio	95% CI	P-value
≤30kｍ	reference			reference		
31-60 km	0.74	0.32-1.71	0.48	0.68	0.29-1.61	0.39
61-90 km	1.14	0.47-2.73	0.77	1.07	0.44-2.59	0.88
91-120 km	0.47	0.17-1.29	0.14	0.44	0.16-1.21	0.11
≥120 km	0.50	0.25-1.01	0.05	0.46	0.27-0.93	0.03

## Discussion

The small number of cases in this study (387 patients) despite the fact that DSGAH is one of the national referral hospital, might be due to the period when this study was carried out was when the referral system in the era of National Health Insurance in Indonesia has been implemented with new regulations. As such, DSGAH has been receiving particularly advanced cases referred from primary or secondary level health facilities. In this study, we observed that travel distance to the referral hospital (TDH) especially of more than 120 km was significantly associated with a longer period from symptom onset to consultation (SO-C) and only SO-C 31-60 days was associated with PVR severity, yet dose-response relationship was not appeared. This might be due to information bias, unevenly distributed samples in each category or some unmeasured confounding factors. The TDH of more than 120 km was significantly associated with 6mo-FA. This finding reflects that travel distance may impact the willingness of the patient to complete their follow-up treatment in addition to other socioeconomic reasons. However, the result obtained in this study demonstrated that TDH of more than 120 km did not prevent 6mo-FA, this might be influenced by the area of origin, its development index, infrastructure and transportation system availability.

As mentioned by Mitry et al., cases from more deprived regions appear to present later with extensive detachments and have significant implications for the final visual prognosis^10)^. Longer period from symptom onset to consultation was associated with PVR severity in our study was also mentioned in previous studies^11, 12)^. Age, gender^11)^ and ethnicity^12, 13)^ strongly affected the incidence of RD. In this study, the male population was predominant as also seen in several studies^11, 12, 14, 15)^. In terms of age, our result was in agreement with Chandra et al., that RD may occur at a younger age in Asians (46.1 years) as indicated in other studies^13, 14, 16, 17)^. The above results, even after adjustment for age and gender in the multivariate analysis, suggest the importance of the impact of distance to the healthcare facility on RD care. In Indonesia, the distance of more than 120 km to the healthcare facility in some areas is a burden especially when the areas are not traversed by an adequate transportation system. Meanwhile, not all residents in the peripheral area have private vehicles or can afford to arrive by plane; some of them have to wait for their family members to drive them. This problem was also mentioned by Kelly et al. and Mattson: in rural areas, great travel distance, less public transportation, and inconvenient transportation schedule could play a significant role^7, 8)^. There is still a tendency to use private vehicles for mobilization within and between cities in Indonesia. During this research period, the development of intercity buses between provinces on the island of Java hasn't been experienced growth and fluctuated^18)^ despite the fact that the Indonesian government has been accelerating infrastructure development in transportation especially the highway construction and railway transportation services improvement^19)^.

The majority of the patients in this study presented late and had their macula off. This was in line with other studies in developing or third world countries, i.e., many RD patients presented late, which varied from about 2 weeks^4, 20)^ more than one month^21, 22)^, and more than 3 months after the onset of symptoms^3, 20)^. As such, eyes with a long RD duration had significantly poorer visual acuity both at the initial and follow-up examinations^23)^. Another study in Southwest Ethiopia showed many things in common with our study in terms of the average travel distance for patients (average travel distance of 87.5 km±120.7 km) and PVR severity significantly associated with delay in presentation^22)^. A study in Kwazulu-Natal revealed that few patients returned for follow-up or re-open, which meant that the definite success rate was uncertain^2)^. This represents variations in the complexity of the cases, facilities, retina specialist distribution, and willingness for the treatment follow-up.

The likely contributory factors in the delayed presentation include patients' personal factors, facilities, and doctors' delays. The patients' factors include long travel distance^24)^, lack of knowledge^3)^ or unfamiliarity of the symptoms^3, 25-27)^, lack of affordability^20, 24)^, lack of health insurance or coverage for the limited procedure^20)^ and lack of awareness^24, 26, 28, 29)^. As mentioned in other studies, patients from peripheral areas might first attend their nearest optometrist^4, 30)^ and consider the elongated distance^4, 24)^ and were referred elsewhere before presenting to the referral center^4, 30)^. While most of our patients arrived late for many reasons including long travel distances, lack of financial and ignorance of symptoms, the distance they would have to travel to the tertiary hospital affects their decision. This includes transportation fees, accommodation expenses, and meal allowance for their companions during treatment, especially when the patients require hospitalization for surgical procedures that may take several days or weeks. Additional sources of late presentation are scarcity of facilities^24)^ and clinical resources^3)^, lack of primary eye care^2, 24)^ and lack of vitreoretinal surgeons^20, 24)^. In this study, patients from peripheral areas, in addition to their cultural backgrounds, prefer to visit their local nurse or general practitioner first at a primary care unit since general ophthalmologists with adequate facilities are also limited. Those considerations might contribute to longer SO-C. Although accurate cut-off for the macula-off duration and RD duration to intervention is difficult to determine, the majority are in agreement that the macula-off duration is inversely proportional to visual recovery^30, 31)^. Therefore, urgent interventions to shorten macula-off RD duration may provide better long-term VA results^32)^.

This study has several limitations in terms of its retrospective nature that comes from single center. During the study period, transition from manual to electronic medical record was applied and contributed to our inability to collect complete sociodemographic data including education level, per capita income, human/area development index and other important information related to patient's delay, doctor's delay or referral system's delay that could have been unmeasured confounding factors to this study. The TDH was determined roughly from the center of the place of origin (where the patients came from) to the referral hospital. The SO-C was obtained only from the patient's subjective report, while not every patient remembered when the symptoms appeared for the first time, as photopsia, floaters, or visual disturbance. The existence of recall bias should be noted. There were several unknown data in PVR grade and macula conditions.

Despite all limitations, this research supports RD epidemiological evidence in a real-world setting and provides an overview of RD in one referral hospital in Indonesia that lacks a vitreoretinal specialist and facilities in certain areas of Indonesia that result in late presentation. In addition to the aforementioned, this study will help us to learn about the availability of facilities for patients that can shorten the referral mechanism, which could benefit patients. Further prospective research regarding risk factors in late RD presentation, whether from the patient's or doctor's delay and scarcity of facilities need to be revealed. For example, a questionnaire regarding the knowledge and awareness of RD patients and their association with final anatomical and functional outcomes should be conducted for evaluating the referral routes and obstacles from the primary care provider to the tertiary eye care unit.

In summary, this study observed that a travel distance of more than 120 km is significantly associated with the longer period from symptom onset to consultation and 6 months follow-up attendance. The aforementioned is significantly associated with PVR severity that may result in redetachment and poor prognosis. In order to improve the final VA outcome, it is essential to refer RD cases in a timely fashion to prevent any delays, especially in macula-on RD. Furthermore, the government needs to enhance access to eye care and develop novel approaches to provide accessible and affordable healthcare in peripheral areas. Additionally, continuing medical training and raising awareness in relation to RD emergency for frontline health workers as well as education for patients or people at risk are expected. Along with the development in social media, the importance of RD as one of ophthalmic emergency could be delivered through webinars and many platforms of social media both for health workers and people at risk.

## Funding

The authors received no financial support for the research.

## Author contributions

The conception and design of the study : SAW, YH. Acquisition of data : SAW, MF. Analysis and interpretation of data : SAW, YH. Writing original draft : SAW. Review and editing critically for important intellectual content : YH, KO. Final approval of the version to be submitted : WS, AM.

## Conflicts of interest statement

The Authors declare that there are no conflicts of interest.
